# Clinical and radiographical evaluation of propolis and thymus vulgaris extracts compared with formocresol pulpotomy in human primary molars

**DOI:** 10.1038/bdjopen.2016.5

**Published:** 2016-07-29

**Authors:** Hesham Alolofi, Manal El-Sayed, Sherine Taha

**Affiliations:** 1 Orthodontic, Pediatric Dentistry & Prevention Department, Faculty of Oral and Dental Medicine, Sana’a University, Sana’a, Yemen; 2 Pediatric Dentistry & Public Health Department, Faculty of Oral and Dental Medicine, Cairo University, Cairo, Egypt

## Abstract

**Objectives/aims:**

This study aimed to examine the success of vital pulpotomy using natural extracts on primary teeth.

**Materials and methods:**

The study was carried out on 60 primary molars in 20 children indicated for pulpotomy. Primary molars were treated with formocresol (20 teeth), propolis ethanolic extract (20 teeth) or thymus vulgaris ethanolic extract (20 teeth). Treated teeth were clinically and radiographically evaluated after 1, 6 and 12 months.

**Results:**

The clinical success of formocresol and propolis groups was 88.2%, whereas the thymus group showed 94.4% with no statistical significance difference. The radiographical success for formocresol and propolis was 73.3%, and thymus was 88.2% without any statistical significance difference detected.

**Conclusion:**

Promising clinical and radiographical success rates of propolis and thymus vulgaris obtained when compared with formocresol.

## Introduction

Dental caries is one of the most prevalent epidemic chronic infectious disease, which is amenable to prevention and treatment at both the individual and population levels.^1^

The process of dental caries is nonstop and may continue especially in children till degradation of the dental hard tissues and infection of the dental pulp.^2^ Infected pulp tissue in primary teeth is usually treated with pulpotomy, which is defined as ‘the amputation of infected coronal pulp, and treatment of the radicular uninflamed tissues with pulpotomy medicaments’.^3^

Despite the new medicaments introduced for pulpotomy over the past years, formocresol pulpotomy still has popularity among paediatric dentists.^4^ Fixative properties, clinical success and availability of formocresol are common factors of its popularity. Nowadays, there are many concerns about the safety of formocresol due to the harmful effects of formaldehyde that led to the change towards safer medicaments for pulp therapy such as mineral trioxide aggregate (MTA) and ferric sulfate.^5–8^ Natural products are efficient, less toxic alternatives and constitute a promising source for medicines and new molecules. Propolis is a yellowish brown, sticky, glue-like resinous substance that honeybees collect from various plant species, when mixed with bee’s salivary secretions it becomes a sticky filler substance termed as ‘Bee glue’.^9,10^

The uses of propolis in dentistry were mentioned in several studies.^11–15^ Its antimicrobial effects on different types of oral bacteria is an area of research. These effects proposed the use of propolis in the field of dentistry such as mouth wash, endodontic disinfectant and wound healing material.^10,16–20^ In addition, propolis had been studied as a direct pulp capping and a pulpotomy agent in the primary teeth of animals.^20–22^

Thymus vulgaris is a perennial sub-shrub with small grey or green leaves. It is native to the Mediterranean region and is cultivated in many countries. Several studies showed that extracts from thyme have antimicrobial, anti-inflammatory and wound healing activities.^23–28^

However, its application as a pulp medicament has not been postulated. Thus, the aim of this study was to compare the clinical and radiographic findings of traditional formocresol pulpotomy technique versus propolis and thymus vulgaris extracts when they are added to zinc oxide powder as vital pulpotomy medicaments.

## Materials and methods

### Participants

Sixty primary molars were selected from 12 boys and 8 girls whose age ranged between 4 and 6 years (mean age was 5.2 years) and divided into three equal groups. They were selected from the outpatient clinic at the Department of Pediatric Dentistry & Public Health, Faculty of Oral and Dental Medicine, Cairo University. The procedure, the potential discomfort and risks as well as possible benefits were fully explained to the parents of the children. Then, an informed consent was obtained and singed from the parent.

The clinical and radiographic inclusion criteria were:

1. Healthy and cooperative child.
2. Teeth showed no clinical evidence of mobility.
3. Teeth had no tenderness to percussion, no swelling or opening sinus.
4. Teeth were restorable with stainless steel crown.
5. Absence of external or internal root resorption.
6. Absence of furcal, periapical radiolucency or widened periodontal ligament space.
7. No more than one-third root resorption detected.

### Technique

After clinical and radiographical examination, the treated teeth were anesthetised using 2% lidocaine with 1:100,000 epinephrine (Lignospan Stander, Septodent, Lancaster, PA, USA) and were isolated with a rubber dam. For all treated teeth, caries removed first with a large sterile slow-speed round bur, then the pulp chamber was opened with a sterile high-speed diamond bur under water irrigation. The coronal pulp was amputated completely with a sterile excavator until the orifice. After pulp amputation, haemostasis was achieved using a sterile saline wet cotton pledget with gentle pressure. If bleeding did not stop after 5 min, the tooth was excluded from the study. Then, the pulpotomised teeth were randomly assigned to formocresol, propolis or thymus vulgaris groups.

#### Formocresol group (control group)

The teeth of this group were treated by applying formocresol (formocresol, Dentsply, Surrey, UK) using a sterile cotton pledget for 3–5 min. After removal of the cotton pledget, a reinforced zinc oxide eugenol base then covered the pulp stumps.

#### Propolis group

The teeth of this group were received freshly prepared mix of zinc oxide powder and drops of propolis ethanolic extract till reaching a paste of suitable consistency (~1: 1 ratio by volume) as a pulp medicament.

#### Thymus vulgaris group

The teeth of this group were received freshly prepared mix of zinc oxide powder and drops of thymus vulgaris ethanolic extract till reaching a paste of suitable consistency (~1: 1 ratio by volume) as a pulp medicament. Then, the pulp chamber was filled with a glass ionomer (Riva, SDI, Bayswater, VIC, Australia) as intermediate restoration. All teeth were restored with stainless steel crowns.

### Follow-up

Sixty teeth were followed up clinically and radiographically (using preapical radiographs) at 1, 6 and 12 months±2 weeks. The outcomes in term of success or failure treatment were determined through:

*Clinical evaluation*:
  1. Pain after treatment.
  2. Tenderness with percussion, swelling (abscess) or fistulation.
  Alveolar bone resorption in the apical and/or furcation area (visible periapical or inter-radicular radiolucency).
  Pathological internal/external root resorption.
*Radiographic evaluation*:
  Alveolar bone resorption in the apical and/or furcation area (visible periapical or inter-radicular radiolucency).
  Pathological internal/external root resorption.

Teeth with clinical and radiographic signs of failure were either treated by pulpectomy or were extracted.^29^

The same investigator and another independent experienced clinician who was blind to the treatment made clinical and radiographical outcomes assessment. Data were statistically analysed with Friedman’s test to compare between the three materials. The significance level was set at *P*⩽0.05.

### Sample size and power calculation

The sample size calculation was based on the results of Huth KC *et al.* ^30^ who reported that total success rate of formocresol after 12 months was (96%). Although for propolis and thymus, no relevant studies were found: so the success rate was assumed to be 50%. Using alpha (*α*) level of 5% and beta (*β*) level of 20%, i.e., power=80%, the calculation resulted in 14 cases. To compensate for dropout, the sample size was increased to 20 cases. Sample size calculation was performed using IBM SPSS SamplePower Release 3.0.1 (IBM Software company USA).

### Randomisation technique

Each tooth was randomly allocated to one of the three techniques using sealed envelope randomisation method.

## Results

Sixty teeth were met the inclusion criteria and allocated to three groups (formocresol, propolis and thymus vulgaris). After 6 months follow-up, one patient with three pulpotomised teeth was dropped out for the reason that the patient did not want to come for more follow-up visits. Fifty-seven teeth were included in the final statistical analysis till the end of the follow-up period.

**Figure 1:**
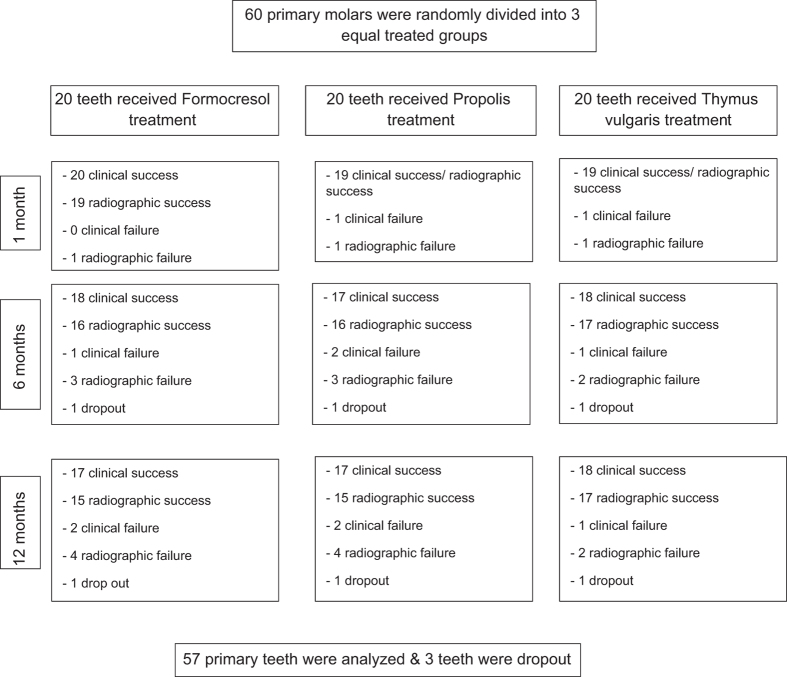
Study design.

Patients’ selection process was summarised as shown in Figure 1.

**Figure 2:**
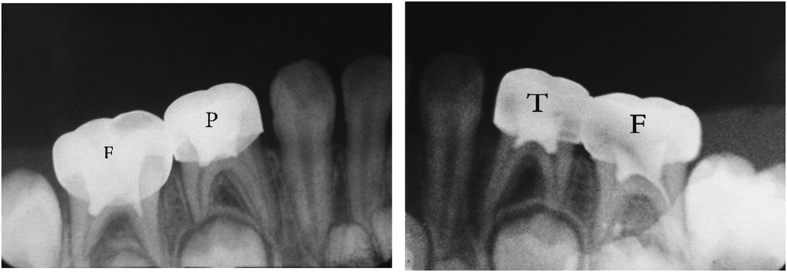
One month post operative.

**Figure 3:**
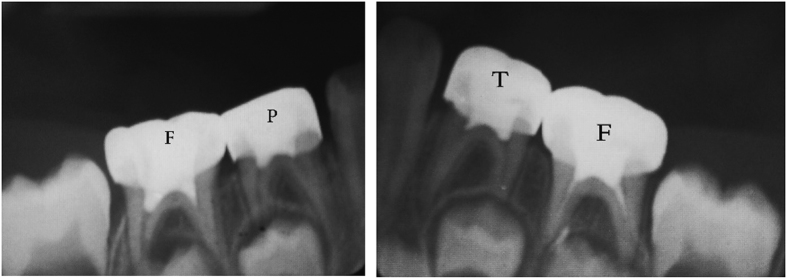
Six months post operative.

**Figure 4:**
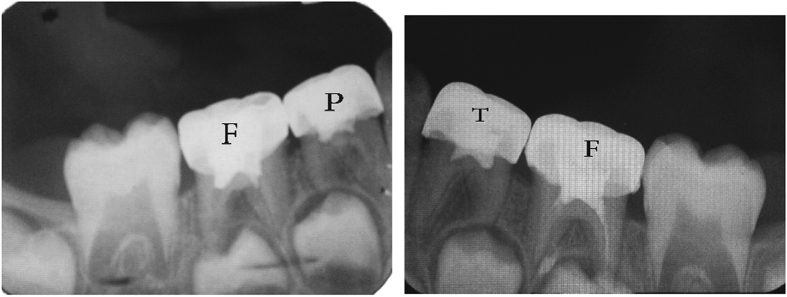
Twelve months post operative.

**Figure 5:**
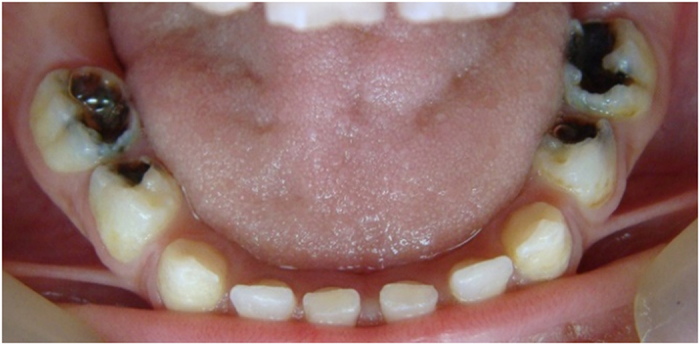
Pre-operative photo.

**Figure 6:**
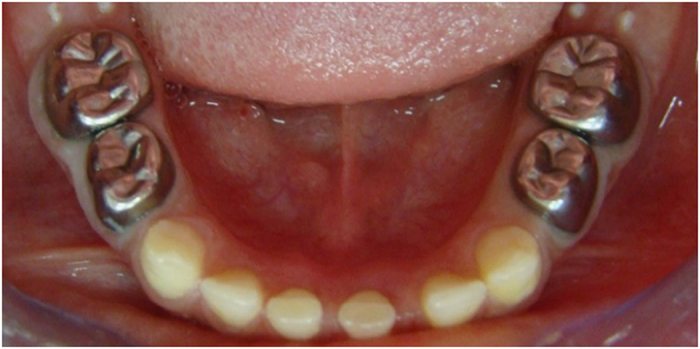
One month after treatment.

**Figure 7:**
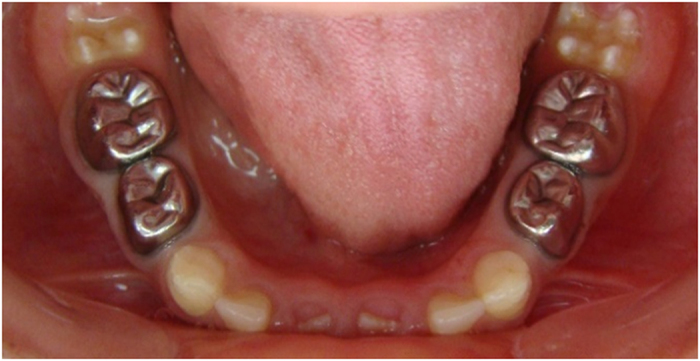
Six months after treatment.

**Figure 8:**
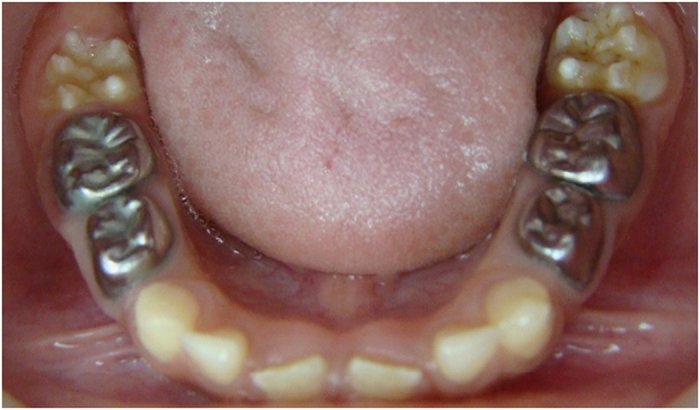
Twelve months after treatment.

The teeth in the formocresol group had a clinical success rate of 100% at 1 month, 94.4% at 6 months and 88.2% at 12 months (Figures 2,3,4,5,6,7,8). The teeth in the propolis group had a clinical success rate of 94.7% at 1 month, and 88.2% at 6 and 12 months. The thymus vulgaris group had a clinical success rate of 94.4% at 1 month, and 94.1% at 6 months and 12 months follow-up. Statistical analysis of the data, using Friedman’s test, revealed no statistically significant differences between the three groups at all follow-up appointments (Table 1).

The teeth in the formocresol group had a radiographic success rate of 95.0% at 1 month, 81.2 at 6 months and 73.3% at 12 months. The teeth in the propolis group had a radiographic success rate of 94.7% at 1 month, 81.2% at 6 months and 73.3% at 12 months. The thymus vulgaris group had a radiographic success rate of 94.4% at 1 month, and 87.5% at 6 and 12 months. Statistical analysis of the data, using Friedman’s test, revealed no statistically significant differences between the three groups at all follow-up appointments (Table 2).

## Discussion

Pulpotomy is a common treatment procedure for cariously exposed pulps in primary teeth. This procedure helps to maintain the integrity of primary teeth that have inflammation limited to coronal pulp. The main goals of this technique are to preserve the radicular pulp, maintain vitality and ultimately to retain the tooth.^31^

Back to nature is one of the recent concepts that takes a great attention in the medical as well as in the dental field. It was found that the use of herbal (natural plants containing biological products) has been increased especially with increasing the resistance of pathogenic microorganisms to the traditional medicines.^32^

Recently, the interest in natural resources for medications and the use of essential oils and extracts of many plant species have become more and more popular. This popularity is derived from their curative properties and their antimicrobial activities that have been recognised for centuries.^33^

In the current study, the authors have faced great challenges regarding the use of different materials and techniques for pulpotomy of primary molars. Regarding the formocresol group, the authors conducted the traditional technique and used a base material of zinc oxide powder and eugenol to seal the amputated pulp. However, in the test groups, they did not use eugenol as a liquid, but the challenge here was to mix the zinc oxide powder with the natural extracts (propolis and thymus vulgaris). Unfortunately, the authors did not find any guiding piece of writing regarding this subject, so by trial and error a paste of suitable consistency was obtained to seal the pulp stumps. In our opinion, replacement of eugenol by the natural extracts may be reflected on the results of this study.

The control group was treated with formocresol, as it is still the gold standard for pulpotomy in primary teeth.^34^ However, study groups were treated with propolis and thymus vulgaris. Zinc oxide powder was selected to be mixed with these materials and placed on the pulp tissue because it is non-irritant to the vital pulp.^35^ The authors did not mix zinc oxide powder with formocresol in the control group to eradicate its harmful effect as previously reported, as well as to decrease the diffusion of formocresol through the pulp cells.^36^

The pulpotomy technique in this investigation was carried out by a single operator. This offers the advantage of a strictly consistent and reproducible technique but the potential disadvantage is that the outcomes may be related to a superior operator rather than a superior technique.^37^

Formocresol-treated teeth showed nearly 88.2% of a clinical success rate at end of 12 months of examination. The high clinical success rate of teeth treated with formocresol reported in this study goes in accordance with the results reported earlier as formocresol was known to be a bactericidal and fixative agent.^34,38–41^

However, radiographical failure was evident in 26.7% of teeth. This result goes in agreement with the previous study of Ibricevic *et al.*,^38^ who concluded that formocresol is able to fix superior parts of the radicular pulp tissue than to stimulate healing. The apical part showed characteristics of inflammation, which in turn increases the probability of periapical changes.

Regarding the formocresol group, one case showed internal root resorption during follow-up period. This result goes in agreement with refs 34,^38^,^42^,^43^, who attributed this result to the use of zinc oxide with eugenol as a sub-base. Zinc oxide and eugenol when placed in direct contact with vital pulp tissue may lead to chronic inflammation, which may cause necrosis of pulp tissue or internal root resorption.^34,38,42–46^

To the best of the authors knowledge, this was the first study in Egypt for the use the propolis or thymus vulgaris extract mixed with zinc oxide powder as pulpotomy medicament and sub-base material on human primary molars with follow-up extended up to 12 months.

In the present study, it was observed that pulpotomised teeth treated with propolis showed 88.2% of a clinical success rate at the end of examination period. This result was in accordance with the previous studies that used propolis as a direct pulp capping material,^20,47^ who explained that the components of propolis such as flavonoids, phenolics, caffeic acid, aromatic acids, esters and other derivatives are responsible for wound healing ability, and antimicrobial effect and anti-inflammatory properties.^27,48^ In addition, flavonoids and caffeic acid helped the immune system by promoting phagocytic activities and stimulating cellular immunity that may inhibit bacterial growth in the treated pulp tissue.^20,47,49–51^

The pulpotomised teeth treated with thymus vulgaris showed 94.1% of a clinical success rate at the end of follow-up examination. This high percentage of success may be attributed to the anti-inflammatory, antibacterial and haemostatic properties of thymus components such as thymol, flavonoids, carvacrol and apigenin.^25,27,52–56^ The authors also explained these results on the basis that thymus vulgaris showed an inhibitory and bacteriostatic effects on ~15 types of Gram-positive and Gram-negative microorganisms, hence the presence of this natural material with its components was reflected both clinically and radiographically in this study.

No statistically significant differences were noticed regarding the tested materials used versus formocresol as gold standard in this study. Therefore, the future will be in favour of natural materials to overcome the well-known drawbacks of chemicals as formocresol used for vital pulp therapy in primary molars.

## Conclusions

According to the new methodology used in this study and based on the clinical and radiographical results, it could be concluded that propolis and thymus offers good clinical and radiographical results when compared with formocresol but with no statistically significant differences.

Recommendations

- The use of propolis and thymus extracts for vital pulp treatment of primary teeth was promising as alternatives to formocresol.
- Different concentrations of propolis and thymus extracts should be studied for these natural materials with a longer follow-up period.
- Clinical and radiographical studies are in need to determine the prospective effect of used materials in pulpotomy procedure on permanent teeth.

## Figures and Tables

**Table 1:** Clinically observed failure for formocresol, propolis and thymus pulpotomies at 1, 6 and 12 months follow-ups

*Group*	*1 month*		*6 months*		*12 months*	
	N	*CF (*n*, %)*	N	*CF (*n*, %)*	N	*CF (*n*, %)*
Formocresol	20	0 (0.0)	18	1 (5.6)	17	2 (11.8)
Propolis	19	1 (5.3)	17	2 (11.8)	17	2 (11.8)
Thymus vulgaris	19	1 (5.3)	18	1 (5.6)	18	1 (5.6)
*P*-value	0.368		0.368		0.368	

Abbreviations: CF, clinical failure; *N*, number of pulpotomised teeth.

**Table 2:** Radiographically observed failure for formocresol, propolis and thymus pulpotomies at 1, 6 and 12 months follow-ups

*Group*	*1 month*		*6 months*		*12 months*	
	N	*RF (*n*, %)*	N	*RF (*n*, %)*	N	*RF (*n*, %)*
Formocresol	19	1 (5.3)	16	3 (18.8)	15	4 (26.7)
Propolis	19	1 (5.3)	16	3 (18.8)	15	4 (26.7)
Thymus vulgaris	19	1 (5.3)	17	2 (11.8)	17	2 (11.8)
*P*-value	NC		0.368		0.135	

Abbreviations: *N*, number of pulpotomised teeth; NC, non-computed; RF, radiographic failure.
